# Marking time in lockdown: heroization and ritualization in the UK during the coronavirus pandemic

**DOI:** 10.1057/s41290-020-00117-8

**Published:** 2020-10-17

**Authors:** Lisa McCormick

**Affiliations:** School of Social and Political Science, The University of Edinburgh, 15 A George Square, Edinburgh, EH8 9LD UK

**Keywords:** Crisis, Discourse, Hero, Liminality, Coronavirus pandemic, Ritual

## Abstract

Realism has predominated in discussions about the coronavirus pandemic where politicians, authorities, and commentators debate over the substance and consequence of scientific facts. But while biology played a crucial role in triggering the pandemic, the resulting crisis developed through a social process. In this paper, I argue that the coronavirus pandemic in Britain was successfully framed as a crisis, but that the ritualization of solidarity normally generated by this meaning was compromised. Through an analysis of media coverage and official statements from the government, I trace the discursive construction of the crisis through the deployment of battle metaphors. Building on this discourse analysis, I show how the symbolic alignment of the pandemic and the Second World War revived symbols and tropes that informed the cultural construction of pandemic heroes. To explain why the intensity of the crisis framing was not matched in ritual performance, I consider how the government’s ambiguous policies and erratic social performance produced a state of indefinite liminality, subverting solidarity processes in lockdown. The paper offers insight into the experience of anomie during the pandemic and contributes to the strong program in cultural sociology by incorporating the crisis approach in disaster studies into the social drama framework.

On 30 January 2020, Dr. Tedros Adhanom Ghebreyesus, Director-General of the World Health Organization, declared a public health emergency of international concern over the global outbreak of novel coronavirus. The figures were alarming. Nearly 10,000 cases of infection had been recorded in China, where extraordinary measures had been introduced by the authorities to restrict travel to and from the most-affected areas. A further 98 confirmed cases had appeared in 18 other countries. While no one had yet died from coronavirus outside China, the WHO was concerned that the virus could spread to countries with weaker healthcare systems, and it issued a series of recommendations to reduce this risk.^1^

In the ensuing months, the epicentre of the pandemic rolled across the globe, international travel all but ceased, country after country went into lockdown, and the number of confirmed cases and deaths worldwide soared. People talked of little but the coronavirus, and the outbreak became almost the only story reported in newspapers and television news broadcasts as regular activities were suspended and economies shut down. Understandably, politicians, authorities, and commentators mainly resorted to realism in their discussions of the situation. The most pressing issues were epidemiological: Where did the infection start? How easily does coronavirus spread? What are the routes of transmission? Does the virus survive on environmental surfaces? How long are the incubation and infectious periods? What are the symptoms? How many of those infected become seriously ill? How lethal is it? Who is more likely to become seriously ill? Are there any treatments? How long will it take to develop a vaccine? The difficulty of establishing a solid scientific understanding of the new virus was further complicated by the circulation of misinformation on social media.

Biology undoubtedly played a crucial role in triggering the pandemic, but the resulting crisis developed through a social process. The facts about the virus did not speak for themselves; indeed, the frequency with which anything virus-related was described as “unprecedented” signalled that making sense of the situation was not straightforward. As the facts emerged, the pandemic came to be interpreted in a way that raised the stakes and created a sense of urgency that demanded an official response and inspired grassroots efforts. In this respect, the coronavirus pandemic resembles other massive disruptions, such as scandals, assassinations, and terrorist attacks, which become transformed through interpretive processes into “riveting moral symbols” that “initiat[e] a long passage through sacred time and space and wrenching conflict between pure and impure sacred forms” (Alexander 2003, p. 155). But this is not to suggest that the successful crisis framing determined the course of a ritual-like process. In this paper, I argue that a state of indefinite liminality emerged during lockdown in Britain, compromising the ritualization of solidarity that usually accompanies a situation that has been defined as a crisis.

To begin, I present the theoretical framework informing my analysis. I review how the strong program has refined Turner’s social drama model and explain how this can be fruitfully combined with the crisis approach in disaster studies. This discussion sets up my analysis of pandemic discourse in the British context, where I trace how the crisis was framed through battle metaphors (e.g. “fight the virus”) which were introduced by government officials and then elaborated in the mainstream news media. My data are drawn from national news outlets (broadsheets, tabloids, magazines, and internet sources) as well as official government statements (*n* = 111).^2^ Therefore, the sacred symbols, cultural codes, and collective memory of England feature most prominently in the analysis.

It important to note that in the UK, health is an issue that is devolved to the four nations of England, Wales, Scotland and Northern Ireland. While there was some coordination across the four nations in the response to the pandemic, each nation set its own regulations, published its own statistics, and produced its own version of public information campaigns. For present purposes, I will elide the fact that Westminster is simultaneously the English counterpart to the regional governments and the national government of the UK and speak of a British response and a British public. It should also be noted that the deployment of militaristic rhetoric in response to the pandemic is hardly unique to the UK. For example, in China, official media outlets branded President Xi the “Commander of the People’s War” against COVID-19^3^; similarly, President Macron used the phrase “we are at war” (*nous sommes en guerre*) no fewer than six times on 16 March 2020 when he announced that France was entering lockdown,^4^ and he declared a “first victory against the virus” when quarantine measures were lifted on 14 June 2020.^5^ However, by focusing on the British case alone, I am able to reveal how the battle metaphor gained considerable traction in the public sphere by symbolically aligning the coronavirus crisis with collective memory of the Second World War.

The next section explores how the revived symbols and tropes from the Second World War informed the cultural construction of pandemic heroes. I examine the sensation surrounding Captain Tom Moore to illustrate the heroization of an individual, as well as the heroization of healthcare workers as a social group. These examples demonstrate how forcefully the battle metaphors resonated in the public sphere. And yet, while the military language provided an effective interpretive framework for making sense of the situation, it did not translate well into action.

After the discussion of pandemic heroes, I explain why ritual performance during the pandemic failed to match the level of intensity demanded by battle metaphors. The British public were initially galvanized into action, but the erratic social performance of political leaders combined with ambiguous national policy undermined solidarity, leaving them marking time in lockdown. To conclude, I reflect on the issue of temporality and the state of indefinite liminality.

## Conceptualizing crisis

The concept of social drama is the best starting point for a cultural sociology of the coronavirus pandemic because it combines three powerful analytic components: interpretation, performance and temporality. The dominant approach draws on Turner’s (1974, 1982) model, which was developed in his study of conflict and power struggles in small- and large-scale societies. Turner divided the social drama into four stages. It begins with a “breach of regular, norm-governed social relations between persons or groups within the same system of relations” (Turner 1974, p. 38). A crisis phase with “liminal characteristics” follows, during which the breach extends “until it becomes coextensive with some dominant cleavage in the widest set of relevant social relations to which the conflicting or antagonistic parties belong” (Turner 1974, p. 38). In the third phase of “redressive action”, adjustive mechanisms are unleashed to limit the spread of the crisis. Liminality persists as “pragmatic techniques and symbolic action reach their fullest expression” in a desperate attempt to “restore peace among the contending groups” (Turner 1974, p. 41). In the final phase, stability is restored either through the reintegration of the warring factions, or through the recognition of an irreparable schism.

In applications of Turner’s ideas, cultural sociologists have corrected the teleological and functionalist tendencies of his model by placing more emphasis on contingency and interpretation. A classic example is Alexander’s (2003, p. 157) analysis of the Watergate scandal, in which interpretation features in nearly all the factors required for “an entire society to experience a fundamental crisis and ritual renewal”. The crisis begins when a significant portion of the population interprets an event as polluting or deviant; it deepens when the pollution is perceived to threaten the symbolic “center” of society; and it concludes when processes of “symbolic interpretation enforce the strength of the symbolic, sacred center of society at the expense of a center that is increasingly seen as merely structural, profane, and impure” (Alexander 2003). While Alexander traced the interpretive process through binary codes, Jacobs (2000) introduced methods of narrative analysis in his study of the Watts uprisings of 1965 and the Rodney King police beating of 1992. Here again, a crisis is found to begin when an event becomes “narratively linked” through journalists’ accounts “to a central cleavage in society and demands the attention of citizens as well as political elites” (p. 9). A separation from ordinary, everyday life occurs once this event is widely perceived to be so much more than an isolated act of deviance that it constitutes a threat to institutional legitimacy and shatters the society’s idealized view of itself (Jacobs 1996).

Although a pandemic does not involve the sort of transgression or violent act typically found in sociological treatments of social drama (see for example Eyerman 2008; Wagner-Pacifici 1986), it can serve as the unexpected and exceptional event that disrupts everyday routines and compels the attention of citizens and political elites. However, it must first be interpreted as a serious breach. Here the Turnerian tradition can be usefully combined with the crisis approach in disaster studies, which defines crisis as “a *threat* that is perceived to be existential in one way or another” (Boin et al. 2018, p. 24, original italics). Objective indicators, such as statistics, do not automatically register as threatening; consensus must be achieved regarding what the indicators mean, and whether the problem requires attention. A range of actors participate in making sense of the situation, including experts, social groups, government officials and the media (Boin et al. 2018, p. 35); drawing on cultural codes, previous experience, and the collective memory, these actors negotiate the meaning of the indicators and the severity of the threat. If the threat is perceived to endanger the integrity of critical infrastructures and core values, it becomes framed as a crisis, marking a separation from ordinary, everyday life. Time becomes compressed, inflating the stakes and the significance of actions taken in the hopes of averting the impending disaster: “the threat is here, it is real and must be dealt with now” (Boin et al. 2018, p. 25). Because a crisis is fundamentally ambiguous, opportunities and possibilities remain open and the consequences of the threat remain uncertain (Boin et al. 2018, p. 24). The combination of uncertainty and ambiguity encourages a “subjunctive mood” (Turner 1982, p. 84), lending the crisis period a liminoid quality.

In the Turnerian tradition, it is not only triggering events that demand interpretation, but also the performances of participants in ritual-like processes. Turner (1982, p. 78) regarded the social drama as an “experiential matrix” from which all genres of cultural performance are derived; cultural sociologists have preserved this insight, while also amending the nostalgic sentiments embedded in Turner’s thought, through the development of social performance theory (Alexander and Mast 2006). A central tenet of cultural pragmatics (Alexander 2004) is that collective rituals have not disappeared from contemporary societies, but they have become more contingent; symbolic action can produce ritual-like effects, but only if the social performance of the ritual leader overcomes the problems of defusion and refusion generated by the differentiation of cultural systems and the complexity of social organization. The difficulty of achieving fused performance, and the centrality of ritual-like effects in contemporary society, has been well-demonstrated by applying cultural pragmatics in the domain of politics. For example, Alexander (2010, p. 10) has shown how presidential campaigns can be understood as exhilarating public dramas in which the “struggle for power becomes theatrical”; if candidates succeed in presenting a compelling performance and becoming a collective representation symbolizing civil society, citizen audiences fuse with political performers and their messages. Meaning becomes attributed to candidates through narratives of crisis and salvation that cast politicians as heroes who transcend ordinary political life by moving “the causes for which they fight from earlier despair to contemporary redemption and on to future glory” (Alexander 2010, p. 65).

My analysis of the coronavirus pandemic in the UK employs cultural pragmatics to reconstruct how various social actors navigated social spaces and public arenas in an effort to define and control the meaning of the situation, whether it is to defuse crisis talk or heighten the sense of emergency. Political leaders are necessarily prominent characters in a pandemic social drama because they are charged with convincing the public to accept their assessment of the threat and support their approach to managing the situation by displaying competence, confidence, and conscientiousness (Boin et al. 2018, p. 33). But while politicians and experts might play central roles, they do not always emerge as heroes. To explain why some political performances failed, and how other social actors became collective representations powerful enough to bridge social divisions, I draw from Alexander’s (2010) approach to the performance of politics.

As Smith and Howe (2015) have argued, postcritical theories of the mass media are a powerful asset in the analysis of social dramas in contemporary industrial societies. Crises and collective rituals are not only mediated through mass media; they are also “mediatized”. Following Cottle (2012, p. 263), I understand mediatization to mean that the media does more than simply report on a crisis or document a collective ritual; rather, they constitute them on a public stage and shape reactions and responses by performatively eliciting “collective sentiments and solidarities on the basis of symbolization and a subjective orientation to what should or ought to be”. Cottle has persuasively shown how the news media participate in the ritualization of catastrophe in their coverage of natural disasters. They help to construct a breach in initial aftermath reports by portraying hurricanes and tsunamis as immoral or amoral actors causing disruption; then they assert “communitas” by running stories about selfless rescuers and generous relief efforts that emphasize solidarity and morality; and finally they help to close the ritual process through the visual presentation and narration of public ceremonies of remembrance (Cottle 2012, p. 269). My analysis of the coronavirus pandemic in the UK reveals that the ritual-like process has not achieved this sense of closure, but I draw from Cottle to show how the news media entered into the crisis by participating in the discursive construction of the threat, evaluating the effectiveness of political performances, celebrating heroic figures and civic-mindedness, and drawing attention to government failures.

Having addressed the composition of my theoretical framework, the empirical discussion can begin. The next section focuses on pandemic discourse in the British context. I show how battle metaphors were used in the construction of the crisis and explain why they resonated so powerfully, setting up later discussions of heroization and ritualization during lockdown.

## Fight the virus: battle metaphors in British pandemic discourse

Militaristic metaphors are commonly used framing devices in scientific and medical discourse, especially in news coverage and government policies concerning harmful organisms such as animals, plants, bacteria or viruses (Larson et al. 2005). Over the course of the twentieth century, governments declared war on pests (McCumber Forthcoming; Winston 1997), and, much to Sontag’s (2002) frustration, on diseases such as cancer, TB, and AIDS. In the early twenty-first century, politicians and journalists deployed military metaphors to make sense of the outbreak of Severe Acute Respiratory Disease (SARS), a framing made even more potent by the pandemic’s coinciding with the Iraq war. In China, after an initial period of secrecy, the SARS awareness campaign featured military language; for example, the Chinese Premier Wen Jiabao was quoted in *The People’s Liberation Army Daily* declaring that “the nation is sure to achieve complete success in the battle” and encouraging medical personnel to maintain their “patriotic spirit” in the “battlefield” (Lu 2008, p. 121). In Singapore, the Deputy Prime Minister Lee Hsien Loong declared the country “officially at war” when the first cases were reported, and reporters took to referring to the Home Affairs Minister Wong Kan Seng as the “Donald Rumsfeld of the SARS wars” (Hudson 2008, p. 170). In Taiwan, Prime Minister Yu Shyi-Kun equated fighting the epidemic with fighting a war and described the virus as an “invisible enemy” (Larson et al. 2005, p. 260). While authorities in Hong Kong were slow to marshal war language, the media made frequent use of disease-as-war imagery; for example, the *Hong Kong Economic Times* urged readers to remain vigilant in holding the “‘two chief battle lines’ of precaution and attack”, and *Ta Kung Pao* insisted that each citizen should “think of themselves as a ‘SARS warrior’ with a duty ‘to stand up and fight against’ the virus” (Baehr 2006, p. 55).

It is not surprising, then, that battle metaphors appeared in discourse concerning the coronavirus pandemic in the UK. What is more puzzling is how they gained so much traction in the British public sphere. Addressing this question requires first examining how politicians, authorities, and journalists used militaristic discourse in the cultural construction of the coronavirus crisis, and how the overtones of this language shifted as the sense of urgency increased.

In the early months of the global pandemic, battle metaphors were employed by the British press in coverage of the outbreak in Wuhan, but they were conspicuously absent when the first confirmed cases of coronavirus in England were reported on 31 January 2020. Even though this news arrived the day after the WHO had declared a global emergency, government officials downplayed the threat; the Chief Medical Officer for England, Professor Chris Whitty, struck a reassuring note, insisting that the National Health Service (NHS) was “extremely well prepared and used to managing infections”.^6^ Battle metaphors became prevalent in British pandemic discourse a month later when the government noticeably changed its assessment of the threat, but the impression displayed was more bluster than alarm bell. Frontpage news on 2 March 2020 was that Prime Minister Boris Johnson would chair a meeting of the government’s Civil Contingencies (Cobra) Committee to sign off on a “battle plan” to “tackle the virus”. In his statement about the meeting, Johnson warned that there was “little doubt that [coronavirus] will present a significant challenge for country”, but he vowed that “the government and the NHS will stop at nothing to fight this virus”, starting with setting up a cross-government “war room” of experts to begin a “public information blitz”.^7^

In his public appearances, the Prime Minister reinforced the impression that the threat was well in hand through displays of confidence. On 3 March 2020, at the same press conference when health officials began stressing the importance of hand hygiene, Johnson was “upbeat”, “boasting” about how he continued to shake hands “with everybody”, including coronavirus patients, during a recent visit to a hospital.^8^ He joked with reporters about taking parental leave when his fiancée, Carrie Symonds, delivered the child they were expecting in late April,^9^ and smirked as he was filmed demonstrating proper hand sanitation on a visit to a research laboratory.^10^ As Johnson sustained his performance of joviality, British citizens responded to the disease-as-war imagery by “stockpiling” supplies as a precaution, emptying supermarket shelves of hand sanitizer, pasta, and toilet paper.^11^

Military metaphors acquired more serious overtones when the government began to describe the coronavirus situation in terms of a crisis. In a “solemn” statement delivered on 12 March 2020 following an emergency meeting with the first ministers of Scotland, Wales and Northern Ireland, Johnson declared that the coronavirus would be “the worst public health crisis in a generation”, and warned the British public that “many more families are going to lose loved ones before their time”.^12^ By specifying that the “most important task” was to “protect our elderly and most vulnerable people”, he indicated that core values, the safety and security of citizens, were endangered. He deferred to the Chief Medical Officer to set out the “lines of defense” which had to be “deploy[ed] at the right time to maximize their effect”, but concluded his statement by reassuring citizens that the country “will get through this epidemic, just as it had got through many tougher experiences before”, referring obliquely to the world wars.^13^

Media coverage reinforced both the military language and the crisis framing. Two days after this announcement, the *Sunday Telegraph* reported that Johnson had put British industry on a “war footing” to equip the NHS for the “coronavirus battle ahead”, calling his request that manufacturers transform production lines to make ventilators an “unprecedented peacetime call to arms”.^14^ The same newspaper published a column by the Health Secretary, Matt Hancock, where he directly compared the coronavirus crisis to the Second World War:

Our generation has never been tested like this. Our grandparents were, during the Second World War, when our cities were bombed during the Blitz. Despite the pounding every night, the rationing, the loss of life, they pulled together in one gigantic national effort. Today our generation is facing its own test, fighting a very real and new disease. We must fight the disease to protect life.^15^

The scale of the impending disaster became clear when Sir Patrick Vallance, the Chief Scientific Adviser for England, told the Commons health select committee on 17 March 2020 that 20,000 deaths would be considered “a good outcome” for the outbreak in the UK.^16^ As the government shifted its attention to protecting critical infrastructures, military language continued to predominate. When Rishi Sunak, Chancellor of the Exchequer, announced a £350bn rescue package to help businesses and workers during the outbreak, Johnson explained that his cabinet “must act like any wartime government and do whatever it takes to support our economy”.^17^ Three days later, the government instructed businesses and entertainment venues to close, inspiring headlines in *The Times* such as “Lockdown for a year is our best weapon against spread”. In describing the response of the Scientific Advisory Group for Emergencies (SAGE) to the question of whether rioting would occur, reporters wrote that experts anticipated that “Britain will […] Keep Calm and Carry On”, referring to the popular propaganda slogan mistakenly associated with the Second World War (Lewis 2012). Military language had even extended to the terminology used to describe social distancing measures; keeping people with vulnerabilities at home was no longer “cocooning”, but “shielding”.^18^

By the time the Prime Minister addressed the nation on 23 March 2020, the meaning of the situation had settled. Even political opponents expressed support for the government’s crisis framing and response; the then Labour party leader, Jeremy Corbyn, posted on social media that “the prime minister is right to call for people to stay at home”.^19^ Battle metaphors lost all earlier traces of playfulness and bombast as Johnson’s performance sobered to declare a “moment of national emergency”. He recognized those who were “working flat out to beat the virus”, including medical staff “on the frontline”, but warned that the NHS was in danger of being overwhelmed. Johnson referred to the coronavirus as an “invisible killer” that could only be overcome through a collective effort: “in this fight we can be in no doubt that each and every one of us is directly enlisted”.^20^ The military forces were also mobilized. The army began delivering food parcels and medicines to “hundreds of thousands of vulnerable people”,^21^ converting conference centers into field hospitals,^22^ and supporting the NHS with deliveries of personal protective equipment and other supplies.^23^

The sense of urgency, and the potency of battle metaphors, was further reinforced when the Queen delivered a rare televised address on 5 April 2020. In the *Daily Telegraph*’s coverage of the speech, reporters wrote that the Queen had “evoked memories of Britain’s Blitz spirit” by comparing the “measures to beat coronavirus to wartime evacuations”, and they noted how she concluded the speech by “echoing the words of Dame Vera Lynn’s wartime anthem” with the promise “we will meet again”.^24^ More than a month later, the UK was still in lockdown, and the Queen addressed the nation again. On this occasion, it was to mark the 75^th^ anniversary of Victory in Europe (VE) Day. Cementing associations between the coronavirus lockdown and the Second World War, the headline in the *Daily Telegraph* read “75 years on, Queen hails ‘strength and courage’ in face of new enemy”, and the reporter’s summary of the Queen’s speech was that she had “urged us all to once more draw on those reserves of national determination to overcome the enemy in our midst”.^25^

The symbolic alignment of the pandemic crisis with the collective memory of the Second World War turbocharged the resonance of battle metaphors in the British public sphere. As Beaumont (2018) has argued, “it scarcely needs stating that a central component in the UK’s national self-narrative is the Second World War”, pointing to the plethora of Second World War films and documentaries broadcast on British television, and English football fans’ predilection for singing songs about winning the war, as evidence of how deeply it permeates the national consciousness. Few myths rival its sacred status in British culture, which is why tropes and metaphors from the Second World War are routinely deployed in discussions about contentious political issues, such as Brexit (Higgins 2020; Tincheva 2019). According to Connelly (2014), the British myth of the Second World War, which is concocted from both popular and official sources, connects with a particular version of British history that features cherished principles and national qualities. As he explains:

Prized and valued in this interpretation are the moments when Britain stood alone and took it on the chin. Interestingly, this is far more attractive to the British than the moment they began to unleash their power. To the average (if there is such a thing) Briton, words such as Dunkirk, Spitfire, Hurricane, Battle of Britain, blitz carry great meaning. They may not have any detailed interest or knowledge of Britain in the Second World War but these words touch a chord in them. But ask someone about the invasion of Italy, the invasion of Germany or the defeat of the U-boat menace and they are far less certain. Britain’s memory of the war is skewed towards the early years of the conflict because this suits Britain’s self-perception: resolute in a crisis and at its best when alone. ‘Taking it’ has been more important than ‘giving it’. (Connelly 2014, p. 14).

The Second World War myth helped make sense of the pandemic situation by providing powerful archetypes of Britishness, especially, being the underdog, “gallantly fighting against the odds”, and building up from disaster to victory (Connelly 2014, p. 15).

Once invoked, the Second World War myth set the standard against which to measure the performances of main protagonists in the pandemic social drama. A reporter in the *Financial Times* was one of many who wrote that Boris Johnson has been given his “Churchill moment”,^26^ and Johnson’s speech announcing lockdown was compared to Churchill’s celebrated “fight them on the beaches” oration, which was delivered shortly after the Dunkirk evacuation.^27^ As an outspoken admirer and biographer of Churchill, Johnson had done much to encourage his comparison with the iconic wartime leader. Appearing alongside the Prime Minister at daily press briefings were civil servants and cabinet ministers whose performances were equally scrutinized by the media. For example, to evaluate the performative success of the entire crisis *dramatis personae*, one *Times* reporter used the trope “having a good (or bad) war”, a postwar euphemism used to indicate discreetly whether someone’s reputation had been enhanced or ruined through their wartime activities.^28^ The revival of symbols and tropes from the Second World War certainly opened up both dramatic possibilities and pitfalls for political performers during the crisis, an issue to which I will return. In the following section, I turn my attention to how the Second World War myth informed the cultural construction of pandemic heroes beyond Whitehall after the crisis had mutated into a disaster.

## Heroization during the pandemic

The sudden and intense popularity of Captain Sir Tom Moore, a Second World War veteran, provides a splendid example of the heroization of an individual during the phase of the coronavirus pandemic when the death tolls in the UK were rising dramatically. In early April 2020, the 99-year-old former tank commander set himself the challenge of walking 100 lengths of his 25-meter garden before his 100th birthday to raise money for NHS Charities Together, progressing at a rate of ten laps per day. Initially, he set a modest fundraising target of £1,000, posting an amateur video on social media urging well-wishers to support his walk. He came to the public’s attention when his story ran in national media outlets. His fundraising target was increased several times, and then abandoned altogether, when his project became international news. By the time he completed his 100^th^ lap on 16 April 2020, Moore had raised more than £18 m from 700,000 people, setting a new record for the Just Giving website.^29^ The final total when he closed his fundraising webpage was nearly £33 m, which had been amassed from over 1.5 m supporters.^30^

Adulation and recognition for Moore rose even faster than the fundraising total. When Moore completed his 100th lap, he was saluted by a guard of honour from the 1st Battalion of the Yorkshire Regiment, the successor to his own wartime regiment.^31^ Buses, trains, dogs and pizzas were named after him, and messages of congratulations poured in from politicians, celebrities and members of the royal family. He was invited to open via videolink one of the Nightingale field hospitals hastily constructed for coronavirus patients,^32^ and he became the oldest person to have a UK No 1 hit single with his cover of “You’ll Never Walk Alone”, recorded for charity with Michael Ball and the Voices of Care Choir.^33^ He awoke on his 100th birthday to the news that he had been appointed an honorary colonel, and that afternoon, he enjoyed a flypast of a Hurricane and a Spitfire that had been arranged to mark the occasion.^34^ He received a personalized birthday card from the Queen, and 150,000 more cards from supporters around the world, many of them franked with the special postmark created by the Royal Mail to honor him.^35^ In a birthday video tribute, the Prime Minister hailed Moore as a “true national treasure”, describing his fundraising efforts as “a beacon of light through the fog of coronavirus”.^36^ Congratulatory signs were erected in his birth town of Keighley, West Yorkshire while the capital awarded him the Freedom of the City of London in a ceremony streamed on YouTube. His nomination for knighthood was rushed through after Downing Street received over 100 petitions calling for him to receive official recognition.^37^ Folk artworks bearing his likeness, from nail sculptures to chainsaw carvings, were auctioned for charity,^38^ and he inspired a legion of imitators, both across the UK and as far away as Ghana, to begin their own fundraising efforts.^39^

In the media coverage of Captain Tom, journalists were not only reporting on his success raising money. They were also, in Cottle’s (2012, p. 263) sense, “mediatizing” the crisis by encouraging solidary sentiments. Stories about Captain Tom’s fundraising success, and the recognition he received, should be understood as expressions of the “public valorization of moral community” that “re-colonizes” the devastation wreaked by disaster (Cottle 2012, p. 269). But Captain Tom’s story was also more than an isolated incidence of selflessness in a difficult time. Media narratives about Captain Tom centred on his purpose, and because his cultural script drew on powerful symbolic elements, the significance of his performance became inflated to that of a full-blown hero. As Alexander explains (2010, p. 64), purpose is central to the meaning of a heroic figure; this goal connects the hero to a greater cause and “defines an arc stretching from the past to the future via the present”, propelling the narrative from despair through redemption and towards glory.

**Fig. 1:**
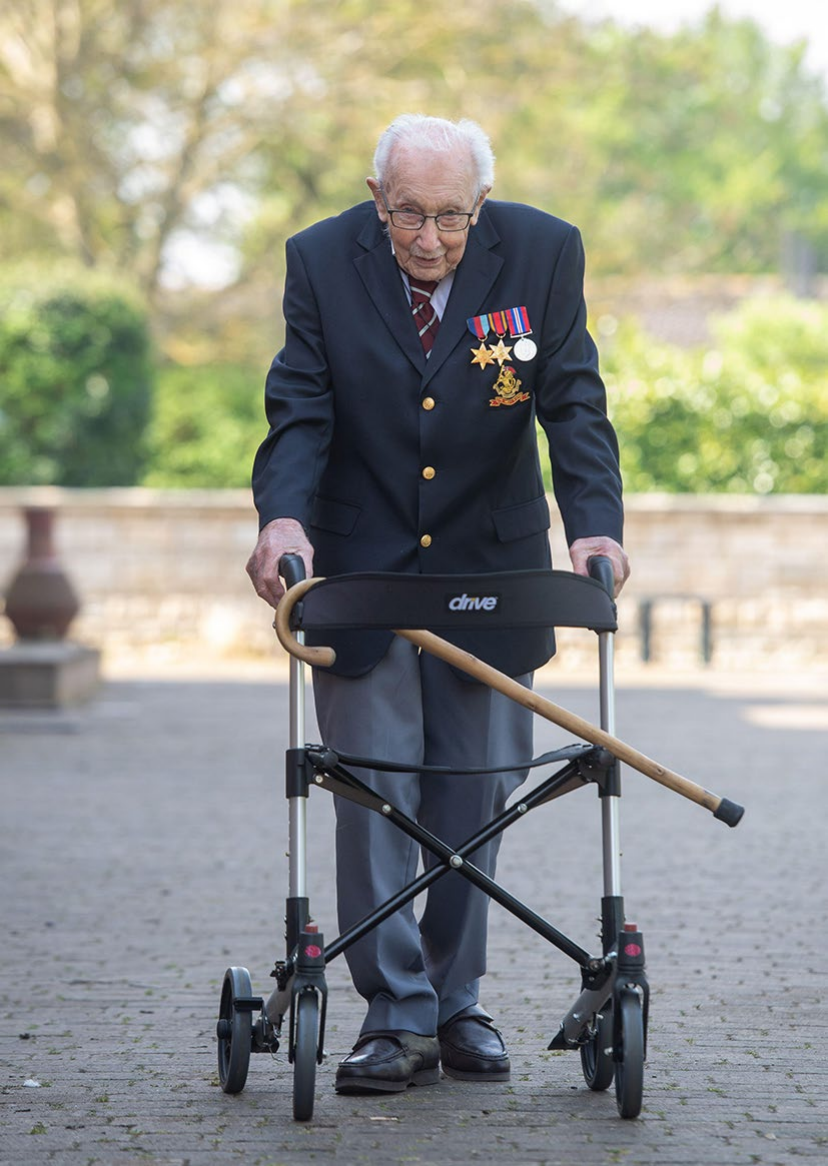
Sir Captain Tom Moore on his charity walk. Photograph by Joe Giddens, PA Images/Alamy Stock Photo

The symbolism of Captain Tom operated on several levels. First, he substantiated the parallel between the Second World War and the coronavirus crisis which had already been interpretively linked in the media; then as now, the country was besieged by a little-understood enemy, and Tom Moore was stepping forward to do his bit for King/Queen and country. As a white male veteran, he was literally a relic from a sacred time in British history and could simply have represented a stereotyped version of cherished national characteristics. Instead, he embodied them through his enactment of the walk for charity. The images of Captain Tom that circulated widely in the media, and then became reproduced in folk artworks, alternated between portraits of him as a young soldier in uniform during the Second World War, and footage of the old soldier setting about his task. The former warmed the nation’s hearts, but the latter set many a stiff upper lip quivering. They showed Moore as a solitary figure smartly dressed in a jacket and regimental tie, his campaign medals glinting in the sun, hunched over his metal walking frame to parade slowly but steadily back and forth behind his home (see Fig. 1). This image was compelling because it personified the country’s response to the worsening situation in a way that fits with British self-perception while also connecting the pandemic with the British version of history described above. Moore was frail, and Britain was being battered by the virus, but these underdogs were resolute in a crisis, and determined to fight gallantly against the odds. As Moore said, echoing the wartime slogans of yore: “Let’s all carry on and remember that things will get better. Where we’ve had problems before, we’ve overcome them, and we shall all overcome the same thing again”.^40^

An important factor in Moore’s heroization was his modesty in public appearances and statements. Alexander (2010, p. 84) has argued that modesty is a crucial element in a hero’s performance because “they cannot be seen as overreaching, as trying to be a hero instead of simply and naturally being one”. In Moore’s account of the charity walk’s origins, he explained that it was originally a “joke, but then it seemed to get bigger and bigger”.^41^ In other words, the success of the charity walk was more happy accident than the result of individual ambition, which would have been both hubristic and un-British. Moore was seen to be unwavering in his dedication to his purpose, never pausing to indulge in self-congratulation; one journalist reported that when he completed his 100^th^ lap, he permitted himself only a “brief nod at a job well done” before vowing to continue walking and raising money.^42^ Even as his celebrity grew, he appeared to shun personal accolades, responding with some embarrassment to calls for him to receive a knighthood: “it would be marvellous to have such an honour, but I don’t expect anything like that”.^43^ Deflecting attention away from himself, he repeatedly expressed his admiration of the public’s generosity, and the “absolute heroes” caring for the sick in the coronavirus wards who he deemed “the equivalent of what we were in the Second World War”.^44^ When he was knighted, Moore’s statement was equal parts humility, graciousness, and patriotism: “I am absolutely overwhelmed. Never for one moment could I have imagined to be awarded with such a great honour. I’d like to thank Her Majesty the Queen, the prime minister and the great British public. I remain at your service”.^45^

Compounding the sacredness of Moore’s veteran status was the collective reverence for the beneficiary of his cause. In Britain, the NHS is more than critical infrastructure. It is a treasured institution; since the British Social Attitudes survey first started measuring popular support for nationalized health care in 1983, it has consistently exceeded 90% (Burki 2018). It is also a powerful institution; Davies (2015, p. 18) has gone as far as arguing that the NHS has largely supplanted the Church of England, the official state religion, by becoming “its own form of life-care system”, encompassing “all aspects of family life” from “cradle to grave”. But most importantly, the NHS has become central to British national identity by appealing to the nation’s self-image as a tolerant and caring society (Burki 2018). In popular historical memory, the NHS, founded in 1948, “appears as the rewarding culmination of the (simplistic) narrative of national sacrifice and austerity” during the Second World War (Baker 2015); it is therefore tightly connected with the Second World War myth described above. Its place in the national narrative, and the scale of Britons’ affection for it, was extravagantly displayed at the opening ceremony for the London 2012 Olympics, the ultimate global stage for performing the nation. Because the NHS is “hardwired into the British DNA”, it has become a sacred cow in British politics; “not taking care of the NHS is akin to [politicians] saying they do not care about society – it loses them elections” (Richard Sullivan quoted in Burki 2018). This combination of political expediency and public veneration of the institution explains how the NHS came to be placed at the heart of the three-part public health slogan that became ubiquitous during the coronavirus pandemic: “Stay home – Protect the NHS – Save Lives”.^46^

While the Captain Tom phenomenon illustrates how individuals were heroized during the pandemic, a similar interpretive process shaped the meaning of social groups. Medical workers provide a striking example. While the discursive practice of referring to medical staff as heroes predated the coronavirus pandemic, this trope was buoyed by the battle metaphors in pandemic discourse. Channel 4 broadcast a television documentary entitled “NHS Heroes: Fighting to Save Our Lives” which “reported on what life is like for doctors and nurses on the COVID-19 front line” using footage medical staff filmed on their smartphones.^47^ The *Sun* newspaper called on readers to support Lord Ashcroft’s campaign “to award brave NHS staff fighting coronavirus with the George Cross medal”, the UK’s “second-most prestigious medal” which was created by King George VI in 1940 to recognize gallantry displayed by bomb-disposal technicians during the Blitz.^48^ The Rugby League arranged for more than 20,000 tickets for the 2021 World Cup in England to be given to NHS and social care “heroes”,^49^ and the Camden Town Brewery launched a new brand of lager called “Camden Heroes” to thank “healthcare heroes” and raise money for charity.^50^ More than 500 artists volunteered to participate in the “Portraits for NHS Heroes” campaign to “honour frontline workers” and “capture” in visual artworks “the personal toll of working on Britain’s hospital wards during the coronavirus outbreak, as well as the dedication that has kept the NHS functioning during the worst medical emergency of recent times”.^51^ Street art appeared across Britain paying tribute to “the NHS warrior”; superhero and medical themes were frequently combined by imposing the Superman emblem on the last letter in the NHS logo.^52^ Superhero references also featured in the artwork Bansky donated to Southampton General Hospital. The painting depicts a young boy kneeling at play next to a wastepaper basket containing discarded Spider-Man and Batman action figures; he proudly holds up a “a new favourite action hero”, a female NHS nurse, who is wearing a surgical mask, an apron emblazoned with the Red Cross, and a cape, her right arm outstretched in the pose of Superman in flight.^53^

The heroization of medical staff was not without controversy. Some felt that the valorized social group should be expanded not just to include non-medical staff in hospitals, but also “key workers” of all kinds, including cleaners and supermarket workers.^54^ Others warned against calling health workers “heroes” because it could cause psychological harm.^55^ Another danger identified with the “language of heroism” was that it could be used to “mut[e] critique and debate: heroes aren’t supposed to complain or speak out about inadequate protective equipment or lack of testing capacity, or to point out what damage years of austerity have done to healthcare provision in the UK”.^56^ At the beginning of the crisis, when infection rates were rising at an alarming rate, several newspapers reported that NHS staff “fear[ed] they would die”^57^ because of the shortage of protective equipment, and that they felt like “cannon fodder”^58^ and “lambs to the slaughter”.^59^ One NHS doctor was so frustrated by the “sentimental distraction” from the causes for NHS staff vulnerability that they blatantly refused to be cast as a hero and challenged military discourse in an anonymous open letter that declared: “I don’t work ‘on the frontline’ because there isn’t one; I’m not in the army and we aren’t engaged in military combat.[…] The NHS is not a charity and it isn’t staffed by heroes”.^60^ Other healthcare workers admitted feeling “deeply uncomfortable with their ‘heroic’ status”, preferring instead to be recognized for their “sense of vocation and professionalism” during the crisis.^61^

Neither was the use of battle metaphors in pandemic discourse without its detractors. One columnist deflated war language by describing it as “platitudes about heroes or battles or victories” that politicians were resorting to in a pathetic attempt to “disguise Britain’s grim current reality”^62^; another journalist declared military language “woefully out of place” in a pandemic and exhorted leaders to “lay off those war metaphors”.^63^ Echoing these concerns, the *Atlantic* presented its “case against waging ‘war’ on the coronavirus”, warning that “leaders invoking battle terminology to galvanize national action risk achieving the opposite”.^64^ In the next section, I consider how the British public responded to the rallying cry to “fight the virus”. While they were initially galvanized into action, solidarity became eroded through the erratic social performance of authorities and the uncertainty generated by ambiguous national policies. This compromised ritualization, leaving the public to mark time in a state of indefinite liminality.

## Ritualization during the pandemic

There are several indications that the government’s crisis framing was widely accepted by the British public. The Prime Minister’s announcement of lockdown restrictions on 23 March 2020 was a major media event, attracting one of the largest television audiences in UK television history.^65^ The following day, the *Independent* reported that there was “almost unanimous support for the coronavirus lockdown measures”, citing a YouGov snap poll indicating that “93 per cent of people support the plan unveiled by the prime minister”.^66^ The main message of the address was to “stay at home”; public gatherings of more than two people were banned, and people could be fined if they left home for any reason other than to acquire basic necessities and medicines, get daily exercise, or travel to do essential work. Despite some initial confusion about what activities were permitted under the new guidance, the public largely complied; the *Financial Times* reported that “data capturing transport and footfall in shops during the first two weeks of strict social distancing measures” revealed “a high level of adherence” to the rules even as the weather improved.^67^

Public space accordingly became vacated by all but those deemed “key workers”. The news media featured “eerie” images of city centres normally bustling with people and traffic abruptly turned into ghost towns.^68^ Reporters shuddered at the “deathly hush” that had descended on city streets,^69^ but acknowledged that deserted railway stations, motorways and high streets were also “evidence of civic-minded abandonment”.^70^ In residential areas, children’s paintings of rainbows appeared in windows as a display of solidarity, a lockdown practice that was believed to have started in Italy.^71^ In the British context, however, the rainbow came to symbolize support for NHS workers,^72^ a meaning that was further reinforced when celebrities posted images of themselves on Instagram sporting the “Thank You NHS” rainbow T-shirt sold to raise money for charity.^73^

The British public also displayed solidarity by answering the government’s call for an “army of volunteers”^74^ to help the NHS. On 24 March 2020, Matt Hancock, the Health Secretary, launched an appeal for 250,000 volunteers to help vulnerable people who were self-isolating. More than 500,000 people signed up within 24 hours. When the number of volunteers exceeded 750,000, the Prime Minister recorded a thank you message in which he declared that the widespread willingness to volunteer “proves there is such a thing as society”, contradicting the infamous dictum of his other iconic predecessor, Margaret Thatcher.^75^

The positive response to the appeal for NHS volunteers provided an ideal focus for media ritualization. Newspapers of every political persuasion seized on it to invoke notions of national community and emphasize the bonds of solidarity forming through adversity (Cottle 2012, p. 268), infusing morality into the scene of unfolding devastation just as it had with Captain Tom Moore. An editorial in the *Times* about the volunteers opened by reminding readers that only “a few weeks ago, Britain’s most pressing problems appeared to be political divisions over Brexit and a spluttering economy”. Communitas, however, was reasserting itself to unite the country and overcome hardship:

…amid these privations [of social distancing measures] a flourishing of civic spirit appears to be taking hold […] It is hugely encouraging that so many are now stepping forward to join a campaign in simple acts of altruism. This is the type of patriotism, outward-looking and generous, that was exemplified by the volunteering during the 2012 London Olympics. Its ethos is recreated now in crisis just as it was exemplified then in celebration.^76^

Moral approbation also abounded in the *Daily Mail*, which praised the “selfless Britons […] stepping forward to help”^77^ by joining the “Corona Home Guard”; in this paper’s view, their enthusiastic response revealed a “public spirit that defies superlatives”, and provided the reassuring “flipside” to that “week’s cataclysmic news”.^78^*The Guardian* slightly dampened mediatization by emphasizing the mundane practical challenges of running the scheme, but the editorial headline nonetheless highlighted “the kindness of strangers”, and the paper’s position was that it was “heartening” that “such proof of altruism” had emerged during difficult times because it suggested that “some good may come out of this crisis”.^79^

While this cultural process seemed to be gathering momentum at the beginning of lockdown, several factors emerged to erode solidarity and interfere with ritualization. First, a central protagonist—the Prime Minister—temporarily disappeared from the stage at a key point the unfolding social drama. Before lockdown measures were introduced, Johnson dominated the daily press briefings from the central podium, seizing these highly publicized occasions to deliver battle discourse first with bravado, and later with gravitas. But five days into lockdown, Johnson was included among the ministers and advisers who had caught the virus.^80^ By 6 April 2020, his illness was severe enough that he was taken to hospital, and on the following night, when the Queen’s speech was broadcast, he was moved to intensive care. Dominic Raab, the Foreign Secretary and First Secretary of the State, was swiftly designated as the deputy “tak[ing] charge of the coronavirus ‘war cabinet’”, remaining in this position for three weeks while Johnson recovered.^81^ Johnson therefore missed a chance to perform a Churchillian script during the country’s darkest hour. But neither was he upstaged by the deputy. Raab was just one of an expanding roster of cabinet ministers taking the daily press briefings,^82^ which diluted the symbolic impact of these appearances.

A second factor hampering ritualization was the government’s erratic and ultimately unconvincing performance of confidence and competence. This weakened the state’s battle script of building from disaster to victory implied by references to the Second World War, allowing social divisions to resurface, and critics to use the battle discourse against the authorities. One damaging blunder was the government’s prolonged inability to deliver on promises to provide virus testing for NHS staff. In early April, ministers admitted that only 0.4% of the NHS workforce had been tested,^83^ and reports from the NHS trusts indicated that a tenth of medical staff were off sick, many of them self-isolating in case they had the virus.^84^ Newspapers across the political spectrum denounced the situation as an “outrage”, “chaos”, a “fiasco”, a “scandal”, and “shambles”.^85^ When the government’s contact tracing schemes encountered similar problems, Sir Keir Starmer, the new Labour leader, accused them of leaving a “huge hole in our defences”.^86^

The government also appeared incompetent because of the shortage of personal protective equipment (PPE), which was forcing NHS trusts to ration supplies,^87^ doctors on intensive care units to reuse visors, and medical staff to make their own gowns out of garbage bags and curtains.^88^ On the frontpage of one tabloid newspaper, the director of a doctors’ group compared medical staff lacking tests and masks to “soldiers being sent to war with no helmets”.^89^ Similarly, a *Times* editorial denounced the government’s “equipment failure”, arguing that “the ability of the NHS, and others on the front line, to win the battle against the pandemic depends on them having access to appropriate personal protective equipment”.^90^ When *The Guardian* reported that the government repeatedly missed opportunities to bulk buy medical supplies through a European Union consortium despite the shortage, political divisions resurfaced through accusations that ministers had made a (pro-Brexit) “political decision not to join the scheme”, and that they were now involved in a cover-up.^91^

As lockdown continued, the list of disappointments lengthened. The antibody testing kits hyped by Johnson as a “game changer” proved to be unreliable.^92^ The contact tracing app that Johnson had pledged would be “world beating” was shelved after a series of mishaps.^93^ Only a fraction of the NHS volunteer army was ever deployed; the “vast majority” of those who reported for duty were never called into action, leaving many volunteers “confused or disgruntled”.^94^ Morale was bruised again when the UK death toll surpassed the symbolic marker of 10,000. The *Times* described the figure as a “cruel test of strategy”, and advisers warned that the UK would have the dubious distinction of becoming “the worst affected nation in Europe”.^95^ Faith in political leaders was further damaged when it was revealed that the government’s chief advisor, Dominic Cummings, had driven from London to Durham during lockdown when he had reason to think he was infected with coronavirus; a special press conference was hastily arranged in an attempt to dampen the fury of the public.^96^

Ambiguous national policy was a third factor undermining ritualization. Newspapers reported that even government supporters were concerned that “mixed messages” were confusing the public, and scientific advisors disparaged the “shifting advice by different ministers” and “apparent contradictions between policies”.^97^ Not only did the government’s plan to reopen society fail to provide any clarity; its unveiling also ruined the coordination between four nations. Following Johnson’s televised address on 10 May 2020, the leaders of Scotland, Wales and Northern Ireland rejected Westminster’s “catastrophic” decision to change the official slogan from “stay home” to “stay alert”, along with their equally “vague and imprecise” guidelines, because they “could jeopardize efforts to control the pandemic”.^98^ One *Times* columnist documented further inconsistencies, contradictions, and complications contributed by cabinet ministers in the days following the “absurd” televised address, and pointed to signs of diminished solidarity:

Slowly but surely, the COVID-19 consensus is breaking down.[…] Although 90 percent of the population supported the introduction of the lockdown, according to a new YouGov survey only 44 per cent support its relaxation with 43 per cent opposed. Chris Curtis, research director of the polling company, says that the ‘rally around the flag’ effect that the prime minister benefitted from at the start of the crisis is coming to an end.^99^

The government had deliberately timed the announcement to coincide with VE day, but rather than provide symbolic reinforcement, the mise-en-scène backfired. According to the government’s own scientific advisor, framing the “partial easing of the lockdown as a victory” was “incredibly unhelpful”, and had left people “justifiably confused” about coronavirus messages.^100^ The lack of clarity about when or how lockdown would ever end only served to reinforce a sense of indefinite liminality.

In these conditions of uncertainty, the mechanical solidarity imposed by lockdown gradually gave way to anomie. When social distancing measures were first introduced, valiant attempts were made to transfer conventional rituals into online environments. The *Times* reported the instant popularity of everything from pub quizzes to choir practices to knitting classes by video call^101^; but as lockdown continued, the enthusiasm faded, and “Zoom fatigue” emerged along with a complete catalogue of hazards associated with video communication platforms.^102^ When the concert season came to an abrupt end, UK-based classical musicians, such as the Kanneh-Mason family, joined their counterparts abroad by posting regular mini-recitals from their living room on social media,^103^ and cultural organizations opened up their digital archives. One *Guardian* journalist took advantage of this “golden age of online arts” by curating her “own festival from the comfort of [her] own home”, but concluded that it was not the same:

[Y]ou make memories at festivals. You meet people who become friends or lovers. There is serendipity and surprise – all this, plus the art. I experienced some of the best art and culture the world has to offer – but without the festival crowds and a posse of friends it’s like the proverbial tree falling in the forest. Did the festival really happen if there was no one else to share it with?^104^

Other ad hoc symbolic gestures of solidarity were introduced, but few were sustained, and none developed into full-fledged rituals. Unlike in Italy, where apartment blocks provided an ideal socially distanced configuration for balcony sing-alongs and concerts,^105^ spontaneous music-making in Britain did not catch on. Where public music performance was attempted, problems were encountered; the *Daily Telegraph* reported that an acclaimed violinist and his family were asked by police to stop providing weekly concerts for neighbours from their front garden because it was “encouraging neighbours to break the lockdown”.^106^

A more successful initiative was Clap for Carers. Following the practice started in other European countries, people stood at their windows or outside their front doors once a week to show their appreciation for essential workers with applause and noisemakers; members of the royal family endorsed the initiative by posting videos and messages of gratitude on social media.^107^ Clap for Carers was also heavily “mediatized” (Cottle 2012). BBC television ended its Thursday News at Ten broadcasts with a series of short clips showing a cross section of society—from young children to the Prime Minister to offshore oilworkers—participating in the ovation from locations across the country.^108^ The *Times* coverage of the first Clap for Carers inflated the meaning further by connecting the gesture with the Second World War myth by paraphrasing Churchill’s famous Dunkirk speech:

They clapped them from the balconies; they clapped them in their living rooms; they clapped them with their memes and with their tweets; they clapped them in their gardens. At 8 pm last night, a fretful nation paused for applause to show its gratitude to those fighting in the front line against the invisible enemy.^109^

But even this form of ritualization was undermined. Clap for Carers ended after its tenth week when the founder joined the growing chorus of calls to cease the practice, citing concerns that it had “had its moment” and was “becoming politicized”.^110^ Rather than join the applause, some healthcare workers used social media to say that they needed personal protective equipment more than they needed applause; others used the occasion to stage protests, including one at Downing Street where a banner was displayed reading “doctors not martyrs”, and a wreath was laid to memorialize the NHS workers who had died from coronavirus.^111^ Questions arose about whether it was “hypocritical” for ministers to join in the applause, and many resented that politicians had reduced it to an empty “show” of support.^112^ Apart from political grievances, another concern was the “creeping clapping fascism” manifested in the “competition to make the most obvious and noisiest display” and the shaming of “non-clappers”.^113^

In marked contrast to the clamor of Clap for Carers, a minute’s silence was held on 28 April 2020. This was the sole ceremony of remembrance to be “enacted within and through the news sphere” (Cottle 2012) during the lockdown. In a direct parallel to Remembrance Day, Britons were invited to pause in silence at 11am to honor the “fallen heroes” and “commemorate those on the frontline who died while keeping us alive”.^114^ Battle metaphors and Second World War references were flaunted in coverage published by the *Daily Telegraph*:

The lowering gunmetal skies reflected the sombre mood below, serving as a reminder that our country is not yet – nowhere near – ready to move forward into those Churchillian broad sunlit uplands. Victory is not yet ours. As we mourn friends and loved ones, those in essential services grieve for comrades-in-arms. War analogies are impossible to escape […] COVID-19 is now an insidious occupying force. It is the unseen enemy within, killing the old, infecting the young, destroying families – and cutting a swathe through the selfless and the courageous who are fighting our battle on the frontline.^115^

The powerful images displayed on newspaper frontpages and in television news video montages showed solemn leaders in places of government and grief-stricken health workers in hospital atriums, which provided a “focus around which an imagined nation was summoned and seemingly united as a moral community in sympathy and grief” (Cottle 2012, p. 270).

The mediatization of this ceremony, however, did not bring a sense of closure to the ritual process, as it did with the natural disasters Cottle studied. The date for the occasion was not selected to mark a decisive turning point in the pandemic, or align with a recognizable anniversary; rather, it had been proposed by health workers’ unions to build on the success of Clap for Carers.^116^ The end of liminality, like lockdown, remained out of sight.

## Conclusion

In this article, I suggested how to marry the Turnerian social drama approach in cultural sociology with the crisis approach in disaster studies to understand how a breach can be perceived even in the absence of violence and transgression. My analysis of the coronavirus crisis highlighted the contingency of interpretive and ritual-like processes. On the empirical level, I detailed how the pandemic came to be defined as a crisis in Britain through the deployment of battle metaphors, and how powerful symbols and tropes from the Second World War were revived. In contrast with previous studies of the discursive construction of pandemics, I found that the overtones of the battle metaphor do not automatically heighten the sense of urgency and can shift registers. Finally, I showed that the militaristic framing infused the cultural construction of pandemic heroes but not the state of liminality during lockdown, which undermined the ritualization of solidarity. The battle metaphors resonated with the public, but the battle script proposed by the state failed when inept political performances spoiled the triumph narrative implied though the invocation of the Second World War myth.

Lurking throughout this discussion was the issue of temporality. While this topic deserves fuller treatment separately, some preliminary thoughts can be offered by bringing together several points made over the course of the discussion. In the social drama approach, the crisis is interpretively separated from ordinary times through the perception of a serious breach. The crisis approach in disaster studies complements this idea by emphasizing the flexible temporality within the crisis. Time became compressed during the coronavirus pandemic in the UK when the threat was perceived to be serious and the battle metaphors were more than posturing. The sense of urgency was further heightened by the speed of the developing situation. As the progression to lockdown gathered pace, advice and information became out of date within hours of being released. But once lockdown restrictions were imposed, time slowed down and became confused. Routine activities were suspended across many domains of social life, removing that which previously shaped days and weeks. The cancellation of regular occasions and mega-events, such as annual conferences, concert seasons, festivals, championship finals, and the Olympic Games in Tokyo, interrupted the larger rhythms of the calendar year. During lockdown, empty city streets were compared to the quiet that descends on Christmas Day or to the traffic levels remembered from decades ago, while the experience of prolonged school and office closures was sometimes described as an endless summer.

The “age of COVID-19” has become a cliché, but it can also be seen as a trope representing the anomie of indefinite liminality. Adding to the difficulty of making plans for an uncertain future is the doubt about how much “normal” can ultimately be restored. This points to a side effect of the battle metaphors and Second World War references invoked to frame the pandemic. According to Pong (2020), war rhetoric helps to manage pandemic anxieties because “temporal uncertainty is at the heart of how one experiences wartime—we assume wartime ends, but we don’t know when or how, so we live in a suspended present”. The state might have succeeded in introducing the interpretive frameworks that made the pandemic meaningful, but it squandered their symbolic power by failing to perform its role in the battle script. As with any war, it is much more straightforward to declare it, than to bring it to a firm conclusion.

## Footnotes

1 Boseley, Sarah. “WHO declares coronavirus a global health emergency”. *The Guardian*. 30 January 2020; (https://www.theguardian.com/world/2020/jan/30/who-declares-coronavirus-a-global-health-emergency). “Coronavirus declared global health emergency by WHO” BBC News. 31 January 2020. https://www.bbc.co.uk/news/world-51318246.

2 The newspapers included in the analysis are: *The Times* of London, *The **Daily Telegraph*, *The* *Sunday Telegraph*, *The* *Guardian*, *The Sun*, the* Independent*, the *Financial Times*, the *Daily Mail*, and the *Mirror*. The magazines included in the analysis are as follows: *The Economist*, the *Atlantic*, and *Pianist*. Internet sources were: the BBC News website, Reuters, Sky News, and www.gov.uk (the United Kingdom public sector information website).

3 “China’s propagandists are trapped by their own rhetoric”. *The Economist*, 5 March 2020. https://www.economist.com/china/2020/03/05/chinas-propagandists-are-trapped-by-their-own-rhetoric.

4 «Nous sommes en guerre»: face au coronavirus, Emmanuel Macron sonne la « mobilisation générale» *Le Monde*, 17 March 2020. https://www.lemonde.fr/politique/article/2020/03/17/nous-sommes-en-guerre-face-au-coronavirus-emmanuel-macron-sonne-la-mobilisation-generale_6033338_823448.html.

5 «Emmanuel Macron annonce une accélération du déconfinement et promet de tirer les « leçons» de la crise». *Le Monde*, 14 June 2020. https://www.lemonde.fr/politique/article/2020/06/14/emmanuel-macron-annonce-une-acceleration-du-deconfinement-et-promet-de-tirer-les-lecons-de-la-crise_6042827_823448.html.

6 Ball, Tom, Charlotte Wace, Chris Smyth and David Brown. “Hunt for contacts of coronavirus – stricken pair in York”. *The Times*. 31 January 2020. https://www.thetimes.co.uk/article/hunt-for-contacts-of-coronavirus-stricken-pair-in-york-dh363qf8k.

7 Wright, Oliver. “Johnson: We'll stop at nothing to tackle virus”. *The Times*. 2 March 2020, pp. 1, 7.

8 Grey, Stephen and Andrew MacAskill. “Special Report: Johnson listened to his scientists about coronavirus – but they were slow to sound the alarm”. 7 April 2020. https://www.reuters.com/article/us-health-coronavirus-britain-path-speci/special-report-johnson-listened-to-his-scientists-about-coronavirus-but-they-were-slow-to-sound-the-alarm-idUSKBN21P1VF.

9 https://news.sky.com/video/coronavirus-i-shook-hands-with-everybody-11948548.

10 “Boris Johnson sings happy birthday while washing his hands”. *The Telegraph*. 6 March 2020. https://www.youtube.com/watch?v=EWfZGdLeDl4.

11 Ellson, Andrew and Ashley Armstrong. “Panic buyers fill trolleys with rice, pasta and hand gel”. *The Times.* 4 March 2020, p. 8.9.

12 Smyth, Chris. “Many more families are going to lose loved ones”. *The Times*. 13 March 2020, pp. 1, 2.

13 Prime Minister’s statement on coronavirus (COVID-19): 12 March 2020. (Transcript of the speech, exactly as it was delivered). https://www.gov.uk/government/speeches/pm-statement-on-coronavirus-12-march-2020.

14 Malnick, Edward. “Boris Johnson puts industry on war footing to equip NHS for coronavirus battle ahead”. *The Daily Telegraph.* 14 March 2020. https://www.telegraph.co.uk/politics/2020/03/14/boris-johnson-puts-industry-war-footing-equip-nhs-battle-ahead/.

15 Hancock, Matt. “We must all do everything in our power to protect lives”. *The Daily Telegraph.* 14 March 2020. https://www.telegraph.co.uk/politics/2020/03/14/must-do-everything-power-protect-lives/.

16 Smyth, Chris. “Top adviser says colleagues at odds over way forward”. *The Times*. 18 March 2020, p. 6.

17 Elliott, Francis. “£350bn bailout launched to save Britain’s economy”. *The Times*. 18 March 2020, p. 1.

18 Smyth, Chris and Tom Whipple. “Lockdown for a year is our best weapon against spread”. *The Times*. 21 March 2020, pp. 8, 9.

19 Elliott, Francis, and Steven Swinford. “You must stay at home”. *The Times*. 24 March 2020, pp. 1, 2.

20 Prime Minister’s statement on coronavirus (COVID-19): 23 March 2020. (Transcript of the speech, exactly as it was delivered.) https://www.gov.uk/government/speeches/pm-address-to-the-nation-on-coronavirus-23-march-2020.

21 Smyth, Chris. “Troops will feed the vulnerable”. *The Times*. 23 March 2020, p. 1.

22 Fisher, Lucy. “Conference centre to be field hospital for 4,000 patients”. *The Times*, 25 March 2020, pp. 8, 9.

23 Fisher, Lucy, Katy Lay, and Harry Shukman. “Army may build field hospitals in conference halls”. *The Times*. 24 March 2020, pp. 10, 11.

24 Furness, Hannah. “Better days will return. We will meet again”. *The Daily Telegraph*, 6 April 2020, pp. 1, 2, 3.

25 Shute, Joe. “75 years on, Queen hails ‘strength and courage’ in face of new enemy”. *The Daily Telegraph.* 9 May 2020*,* pp. 2, 3.

26 Barber, Tony. “Boris Johnson’s Churchill moment”. *The Financial Times*. 17 March 2020 (https://www.ft.com/content/a28a70ca-e971-4741-a6cd-277e48115330).

27 Hyde, Marina. “Johnson’s ‘fight them on the beaches’ moment: true grit, or more shifting sands?” *The Guardian*. 24 March 2020. https://www.theguardian.com/commentisfree/2020/mar/24/johnson-fight-them-on-the-beaches-churchill-address.

28 Parris, Matthew. “Who’s having a good or bad war on Covid?” *The Times*. 16 May 2020, p. 21.

29 “We Salute You; Britain can be proud of its charitable response to Captain Tom Moore’s superb efforts”. *The Times*, 17 April 2020, p. 23.

30 “Captain Tom Moore raises nearly £33 m as NHS appeal closes”. BBC News, 1 May 2020. https://www.bbc.co.uk/news/uk-england-beds-bucks-herts-52498156.

31 “We salute you; Britain can be proud of its charitable response to Captain Tom Moore’s superb efforts”. *The Times*. 17 April 2020, p. 23.

32 “Coronavirus: Capt Tom Moore opens Harrogate NHS Nightingale hospital”. BBC News, 21 April 2020. https://www.bbc.co.uk/news/uk-england-york-north-yorkshire-52368691.

33 Snapes, Laura. “Captain Tom Moore becomes oldest artist to claim UK no 1 single”. *The Guardian.* 24 April 2020. https://www.theguardian.com/music/2020/apr/24/captain-tom-moore-becomes-oldest-artist-to-claim-uk-no-1-single.

34 Wilford, Greg. “Capt Tom is flying high as he turns 100”. *The Times*. 1 May 2020.

35 “Coronavirus: Captain Tom Moore gets Royal Mail birthday postmark”. 27 April 2020. https://www.bbc.co.uk/news/uk-england-beds-bucks-herts-52433007.

36 “Sir Tom Moore: 100-year-old fundraising veteran ‘overawed’ to be given a knighthood”. *The Daily Telegraph*. 20 May 2020. https://www.telegraph.co.uk/news/2020/05/19/sir-tom-moore-100-year-old-fundraising-veteran-given-knighthood/.

37 Low, Valentine. “Arise Sir Tom: latest honour for veteran who raised £33 m”. *The Times,* 20 May 2020, p. 4.

38 “Artwork made of thousands of nails honours Capt Tom Moore”. BBC News, 29 May 2020. https://www.bbc.co.uk/news/av/uk-england-leeds-52856247/artwork-made-of-thousands-of-nails-honours-capt-tom-moore; Captain Tom Moore: Chainsaw carving raises £7,000. BBC News, 9 May 2020. https://www.bbc.co.uk/news/uk-england-52574168.

39 “Captain Tom Moore: NHS fundraiser ‘lifted lockdown blues’”. BBC News. 30 June 2020. https://www.bbc.co.uk/news/uk-england-beds-bucks-herts-53178431; “Queen to honour Ghana’s fundraising WW2 veteran Pte Joseph Hammond”. BBC News, 19 June 2020. https://www.bbc.co.uk/news/world-africa-53107602.

40 Coronavirus: Army veteran Tom Moore, 99, raises £4 m for the NHS. BBC News. 15 April 2020. https://www.bbc.co.uk/news/uk-england-beds-bucks-herts-52278746.

41 “Captain Tom, 99, raises $10 million with a walk in his UK garden”. https://www.reuters.com/article/health-coronavirus-britain-veteran/update-1-captain-tom-99-raises-10-million-with-a-walk-in-his-uk-garden-idUSL5N2C36TS.

42 Shute, Joe. “One small step for ‘sir’ Tom, one giant lift for the NHS”. *The Daily Telegraph*. 17 April 2020, p. 5.

43 Ibid.

44 Ibid.

45 Low, Valentine. “Arise Sir Tom: latest honour for veteran who raised £33 m”. *The Times,* 20 May 2020, p. 4*.*

46 Hope, Christopher. “The rallying cry that may have worked too well”. *The Daily Telegraph*, 1 May 2020, p. 14.

47 Singh, Anita. “The terrible reality of COVID-19′s NHS front line”. *The Daily Telegraph*, 7 May 2020, p. 30.

48 Rollings, Grant. “NHS Heroes: Back our campaign to award brave NHS staff fighting coronavirus with the George Cross”. The Sun, 16 April 2020. https://www.thesun.co.uk/news/11415317/coronavirus-nhs-staff-george-cross/.

49 “Free World Cup tickets for NHS and social care ‘heroes’” *The Times*. 27 March 2020, p. 58.

50 “The Good News Drink to Heroes”. *The Daily Telegraph,* 30 April 2020*,* p. 9.

51 R.C. “To honour frontline workers, artists are painting their portraits”. *The Economist*, 10 June 2020.

52 “Street art thanks NHS staff for tackling coronavirus”. BBC News. 23 April 2020. https://www.bbc.co.uk/news/uk-england-52381213.

53 Gompertz, Will. “New Banksy artwork appears at Southampton hospital”. BBC News. 6 May 2020. https://www.bbc.co.uk/news/entertainment-arts-52556544.

54 Jones, Owen. “The undervalued heroes of the coronavirus crisis need our thanks – and our support”. *The Guardian*, 21 March 2020.

55 Petter, Olivia. “Coronavirus: Don’t call NHS workers ‘heroes’, says new mental health guide”. *The Independent.* 30 April 2020. https://www.independent.co.uk/life-style/coronavirus-nhs-mental-health-workers-heroes-a9492341.html.

56 Higgins, Charlotte. “Why we shouldn’t be calling our healthcare workers ‘heroes’”. *The Guardian*. 27 May 2020. https://www.theguardian.com/commentisfree/2020/may/27/healthcare-workers-heros-language-heroism.

57 Lay, Kat. “Doctors fear they will die for lack of safety equipment”. *The Times*. 18 March 2020, p. 9.

58 Bodkin, Henry. “No 10 sends millions of gowns, gloves and masks to NHS workers”. *The Daily Telegraph*. 30 March 2020, p. 4. See also Siddique, Haroon and Sarah Marsh. “NHS staff feel like ‘cannon fodder’ over lack of coronavirus protection”. *The Guardian*. 22 March 2020. https://www.theguardian.com/society/2020/mar/22/nhs-staff-cannon-fodder-lack-of-coronavirus-protection.

59 Crowson, Isaac. “NHS staff are like lambs to the slaughter”. *The Sun*. 23 March 2020, p. 6.7.

60 Anonymous. “I’m an NHS doctor – and I’ve had enough of people clapping for me”. *The Guardian*. 21 May 2020. https://www.theguardian.com/society/2020/may/21/nhs-doctor-enough-people-clapping.

61 Higgins, Charlotte. “Why we shouldn’t be calling our healthcare workers ‘heroes’”. *The Guardian*. 27 May 2020. https://www.theguardian.com/commentisfree/2020/may/27/healthcare-workers-heros-language-heroism.

62 Hyde, Marina. “The horror of coronavirus is all too real. Don’t turn it into an imaginary war”. *The Guardian*, 7 April 2020. https://www.theguardian.com/commentisfree/2020/apr/07/horror-coronavirus-real-imaginary-war-britain.

63 Tisdall, Simon. “Lay off those war metaphors, world leaders. You could be the next casualty”. *The Guardian.* 21 March 2020. https://www.theguardian.com/commentisfree/2020/mar/21/donald-trump-boris-johnson-coronavirus.

64 Serhan, Yasmeen. “The Case Against Waging ‘War’ on the Coronavirus”. *The Atlantic*, 31 March 2020. https://www.theatlantic.com/international/archive/2020/03/war-metaphor-coronavirus/609049/.

65 Stubley, Peter. “Coronavirus: Boris Johnson’s lockdown announcement watched by 27 m people to become one of UK’s most-viewed TV events”. *The Independent*, 24 March 2020, Media, p. 2.

66 Stone, Jon. “Coronavirus: UK lockdown supported by 93 per cent of public, poll finds”. *The Independent*. 24 March 2020, UK Politics, version: 3.

67 Payne, Sebatstian and John Burn-Murdoch. “UK public largely follows lockdown rules – except for Easter break”. 14 April 2020. https://www.ft.com/content/3a654170-d53c-4efa-862a-027496fb6289.

68 See for example Kindred, Alahna. “Eerie photos show London’s empty streets and pubs on first Saturday night of coronavirus lockdown”. *The Sun*. 29 March 2020. https://www.thesun.co.uk/news/11279840/londons-streets-coronavirus-lockdown/.

69 Low, Valentine, Will Humphries and Neil Johnston. “Bustling streets of London fall silent as cities shut down”. *The Times*. 18 March 2020. https://www.thetimes.co.uk/article/silent-streets-as-bustling-capital-empties-and-cities-shut-down-bnsngmx3r.

70 Ronay, Barney. “An unreal city: lockdown in London”. *The Guardian.* 24 March 2020. https://www.theguardian.com/world/2020/mar/24/an-unreal-city-coronavirus-lockdown-in-london.

71 Street, Sam. “Why are people putting rainbow pictures in their windows?” *The Sun*. 2 April 2020. https://www.thesun.co.uk/news/11309096/rainbow-pictures-windows-coronavirus-uk-lockdown/.

72 “Coronavirus: Rainbow posters from around the country show how children pay tribute to NHS workers”. *The Independent.* 8 April 2020. https://www.independent.co.uk/life-style/coronavirus-nhs-nightingale-hospital-rainbows-art-drawing-painting-a9452806.html.

73 Clark, Laren. “Where can I buy a ‘Thank You NHS’ rainbow t-shirt loved by celebs including Cheryl and Laura Whitmore?” *The Sun*. 22 May 2020. https://www.thesun.co.uk/fabulous/11684214/where-buy-thank-you-nhs-rainbow-t-shirt-cheryl-laura-whitmore/.

74 Smyth, Chris and Rhys Blakely. “Call for army of volunteers”. *The Times.* 25 March 2020, pp. 1, 2.

75 Wright, Oliver and Steven Swinford. “This proves there is such […]”. *The Times*. 30 March 2020, pp. 4, 5.

76 “Trumpet Voluntary”. *The Times*. 26 March 2020, p. 29.

77 “Selfless Britons Who are Stepping Forward to Help”. *Daily Mail.* 26 March 2020.

78 Hardman, Robert. “The Corona Home Guard”. *Daily Mail*. 28 March 2020.

79 “The Guardian view on COVID-19 volunteers: the kindness of neighbours; Huge public support for health and care workers gives reason to hope that some good may come out of this crisis”. *The Guardian*. 27 March 2020, Opinion.

80 Wright, Oliver and Steven Swinford. “Raab steps up to take charge of nation’s fight against virus”. *The Times*. 7 April 2020, pp. 4, 5.

81 Swinford, Steven, Oliver Wright and Katie Gibbons. “PM in intensive care”. *The Times*. 7 April 2020, p. 1.

82 Of the 92 briefings held, Matt Hancock, the Health Secretary, made 26 appearances, which is more than any other cabinet minister. In comparison, Dominic Raab led 12 briefings. See “Coronavirus: Daily Downing Street press conference scrapped”. *BBC News.* 23 June 2020. https://www.bbc.co.uk/news/uk-politics-53155905.

83 Sample, Ian and Rowena Mason. “Just 2,000 Key NHS staff have been tested, UK government admits”. *The Guardian*. 1 April 2020. https://www.theguardian.com/world/2020/apr/01/nhs-rules-hampering-coronavirus-testing-drive-say-scientists.

84 Lay, Kat. “Coronavirus: Tenth of medical staff are thought to have gone sick”. *The Times*. 1 April 2020, p. 8.

85 McFarlane, Andy. “Coronavirus briefing: NHS staff testing ‘scandal’ and harrowing survival tales”. *BBC News.* 2 April 2020. https://www.bbc.co.uk/news/uk-52127152; “Newspaper headlines: Hospital building ‘heroes’ and testing ‘shambles’. *BBC News*. 2 April 2020. https://www.bbc.co.uk/news/blogs-the-papers-52130211.

86 Buchan, Lizzy. “Boris Johnson vows test and trace scheme will be in place by 1 June after Keir Starmer presses him over delay”. *The Independent*. 20 May 2020. https://www.independent.co.uk/news/uk/politics/coronavirus-test-trace-boris-johnson-keir-starmer-pmqs-latest-a9523856.html.

87 Gibbons, Katie. “Nurse on a ventilator after staff pleaded for protection”. *The Times*. 3 April 2020, p. 10.

88 Courea, Eleni. “Coronavirus: Doctors are forced to reuse visors as NHS PPE shortages bite”. *The Times.* 13 April 2020, pp. 6, 7.

89 McFarlane, Andy. “Coronavirus briefing: NHS staff testing ‘scandal’ and harrowing survival tales”. *BBC News.* 2 April 2020. https://www.bbc.co.uk/news/uk-52127152.

90 “Equipment failure”. *The Times*. 14 April 2020, p. 27.

91 Wintour, Patrick and Daniel Boffey. “UK government accused of cover-up over EU scheme to buy PPE”. *The Guardian.* 22 April 2020. https://www.theguardian.com/politics/2020/apr/22/uk-government-accused-of-cover-up-over-eu-scheme-to-buy-ppe.

92 Devlin, Hannah. “Coronavirus ‘game changer’ testing kits could be unreliable, UK scientists say”. *The Guardian.* 5 April 2020. https://www.theguardian.com/world/2020/apr/05/coronavirus-testing-kits-could-be-unreliable-uk-scientists-say.

93 Cellan-Jones, Rory. “Coronavirus: What went wrong with he UK’s contact tracing app?” *BBC News*. 20 June 2020. https://www.bbc.co.uk/news/technology-53114251.

94 Butler, Patrick. “NHS coronavirus crisis volunteers frustrated at lack of tests”. *The Guardian*. 3 May 2020. https://www.theguardian.com/world/2020/may/03/nhs-coronavirus-crisis-volunteers-frustrated-at-lack-of-tasks.

95 Swinford, Steven. “Death toll passes 10,000 in cruel test of strategy”. *The Times*. 13 April 2020, p. 10.

96 Kenber, Billy. “I tried to do the right thing but reasonable people may disagree”. *The Times*. 26 May 2020, p. 4.

97 Donnelly, Laura. “’Mixed messages’ on coronavirus lockdown creating public distrust, adviser warns”. *The Daily Telegraph.* 28 April 2020. https://www.telegraph.co.uk/news/2020/04/28/mixed-messages-coronavirus-lockdown-creating-public-distrust/; Mason, Rowena and Ian Sample. “‘Mixed messages’: UK government’s strategy fuels fears of rule-breaking”. *The Guardian*. 7 May 2020. https://www.theguardian.com/world/2020/may/07/mixed-messages-uk-governments-strategy-fuels-fears-of-rule-breaking.

98 Wright, Oliver. “We’ll stick with our stay at home plea, says Sturgeon”. *The Times*. 10 May 2020, p. 5.

99 Sylvester, Rachel. “Boris Johnson is trying to blind us with science”. *The Times.* 12 May 2020, p. 21.

100 Whipple, Tom, and James Shield. “Public baffled by victory messages, claims adviser”. *The Times.* 14 May 2020, p. 7.

101 “Cocktails, choirs and knitting by video call”. *The Times*. 28 March 2020, pp. 12, 13.

102 Rigby, Rhymer. “Look me in the eye and tell me you don’t suffer Zoom fatigue”. *The Times*. 1 May 2020, p. 37.

103 Palmer, Ellie. “Lang Lang delights with live-stream performance of Debussy in New York”. *Pianist Magazine*. 7 July 2020. https://www.pianistmagazine.com/news/lang-lang-delights-with-live-stream-performance-of-debussy-in-new-york/.

104 Delaney, Brigid. “Arts in lockdown”. *The Guardian*. 21 April 2020, Life and Style section.

105 Thorpe, Vanessa. “Balcony singing in solidarity spreads across Italy during lockdown”. *The Guardian*, World News.

106 Carpani, Jessica and Robert Mendick. “No thank you for the music, police tell family quartet”. *The Daily Telegraph.* 30 April 2020, p. 7.

107 “Clapping for Carers”. *The Times*. 28 March 2020, p. 31.

108 “Clap for Carers: UK applauds NHS staff and keyworkers”. BBC News. 2 April 2020. https://www.bbc.co.uk/news/av/uk-52143223/clap-for-carers-uk-applauds-nhs-staff-and-key-workers.

109 Kidd, Patrick. “Applause for carers ripples across the land”. *The Times*. 27 March 2020, pp. 4, 5.

110 “Coronavirus: Clap for Carers should end, says founder”. *BBC News.* 22 May 2020. https://www.bbc.co.uk/news/uk-52773032#:~:text=The%20UK's%20weekly%20applause%20for,annual%20event%2C%20Annemarie%20Plas%20said.

111 Merrifield, Ryan. “Silent ‘doctors not martyrs’ protest held outside No10 during Clap for Carers”. *The Mirror*. 29 May 2020. https://www.mirror.co.uk/news/uk-news/silent-doctors-not-martyrs-protest-22105283.

112 Faulkner, Doug. “Coronavirus: Is this the final hurrah for clap for carers?” *BBC News.* 28 May 2020. https://www.bbc.co.uk/news/uk-52818869.

113 Anonymous. “I’m an NHS doctor – and I’ve had enough of people clapping for me”. *The Guardian*. 21 May 2020. https://www.theguardian.com/society/2020/may/21/nhs-doctor-enough-people-clapping.

114 Woods, Judith. “Beneath gunmetal skies, the nation paused to honour our fallen heroes”. *The Daily Telegraph.* 29 April 2020, p. 5.

115 Ibid.

116 Badshah, Nadeem. “Unions call for minute’s silence for key workers killed by COVID-19 in UK”. *The Guardian*. 20 April 2020*.* https://www.theguardian.com/society/2020/apr/20/unions-call-minutes-silence-key-workers-killed-COVID-19-uk.
